# Behçet's Disease and Aneurysms: A Case Series of Vascular Involvement and Outcomes

**DOI:** 10.7759/cureus.72429

**Published:** 2024-10-26

**Authors:** Mamoona Shah, Ushna Khan, Nitasha Saleem, Talha Kareem, Hassan Ali Saoud Al-Thani, Omer Ehsan

**Affiliations:** 1 Internal Medicine, Hull Royal Infirmary, Kingston upon Hull, GBR; 2 Surgery, Shifa International Hospital, Islamabad, PAK; 3 General Surgery, Hamad General Hospital, Doha, QAT; 4 General Surgery, Nishtar Hospital, Multan, PAK; 5 Vascular and Trauma Surgery, Hamad General Hospital, Doha, QAT; 6 Vascular Surgery, Shifa International Hospital, Islamabad, PAK

**Keywords:** aneurysms, behchet’s disease, peripheral arterial aneurysms, pseudoaneurysm, vasculitis

## Abstract

Large vessel vasculitis (LVV) is a group of inflammatory diseases that affect the aorta and its major branches, causing stenosis, aneurysms, or dissections. LVV can be associated with various disorders, such as IgG4-related disease, Takayasu arteritis, giant cell arteritis, and Behcet’s disease. Aneurysms due to Behcet’s disease are rare and challenging to manage, as they have high rates of morbidity and mortality. Endovascular repair combined with immunosuppression has emerged as a preferred option over open surgery in recent years. However, open repair may still be indicated in some cases where endovascular repair is not feasible or available. We present a case series of four patients with LVV and coexisting aneurysms who underwent surgical repair at tertiary care hospitals in Islamabad and Qatar. Our aim is to demonstrate the clinical presentation, diagnosis, management, and outcomes of these patients and to emphasize the importance of early involvement of vascular surgeons in the care of LVV patients.

## Introduction

Vasculitides are multisystem diseases of the blood vessels affecting multiple organs. They cause stenosis or aneurysms of blood vessels, occasionally mandating early treatment. They are categorized according to the size of the affected vessels. Vasculitis, which involves the aorta and/or its major branches, is termed large vessel vasculitis (LVV) [1,2]. LVV (particularly IgG4-related disease Takayasu arteritis, and giant cell arteritis) is related to aortitis resulting in the formation of aneurysms, calcifications, and even aortic dissections making it imperative that physicians should be well aware of these presentations and on their guard [3].

Behcet’s disease is a multisystemic vasculitis disorder, affecting both venous and arterial trees, and is characterized by an auto-immune mediated inflammatory process of blood vessels [4]. Behcet's disease is common in the Mediterranean and the Far East with the highest prevalence rates seen in certain areas of Turkey. It most commonly affects young adults with males having poorer prognosis and higher mortality rates [5]. Bechet’s disease presenting with arterial involvement has a higher propensity toward aneurysms than thrombosis [6]. Of these patients, a mere 1.5 to 2.3 percent are symptomatic [6,7]. Hence, it is very rare for vascular surgeons to encounter cases of aneurysms associated with Behcet's disease. Multiple studies have reported high morbidity and mortality postoperatively with incidences of anastomotic pseudoaneurysm at the site of anastomosis [8]. Recent data suggests that minimally invasive endovascular procedures combined with immunosuppressive therapies provide better prognosis of disease than open surgical repair [9].

Here we present a series of patients presenting with LVV with coexisting aneurysms in tertiary care hospitals in Islamabad and Qatar. Our aim is to show that open repair, though getting out of favor, still offers salvage in settings where endovascular repairs are not feasible. We also hope that this case series enables the physicians to be on guard and involve vascular surgeons as early as possible while managing LVV.

This is a retrospective case series. The data was collected from online medical records after approval from the IRB committee of Shifa International Hospital (Reference Number: 250-24).

The data collected shows four patients of LVV with associated aneurysms presented at tertiary care hospitals in Islamabad and Qatar between January 2021 and December 2023. Three of these patients had aortic aneurysm and presented in the emergency department (ED). One patient had an aneurysm of the profunda femoris artery and presented in the outdoor patient department. CT angiography was done for all the patients. After confirmation of the diagnosis, all these patients underwent surgical repair.

## Case presentation

Case 1

**Figure 1:**
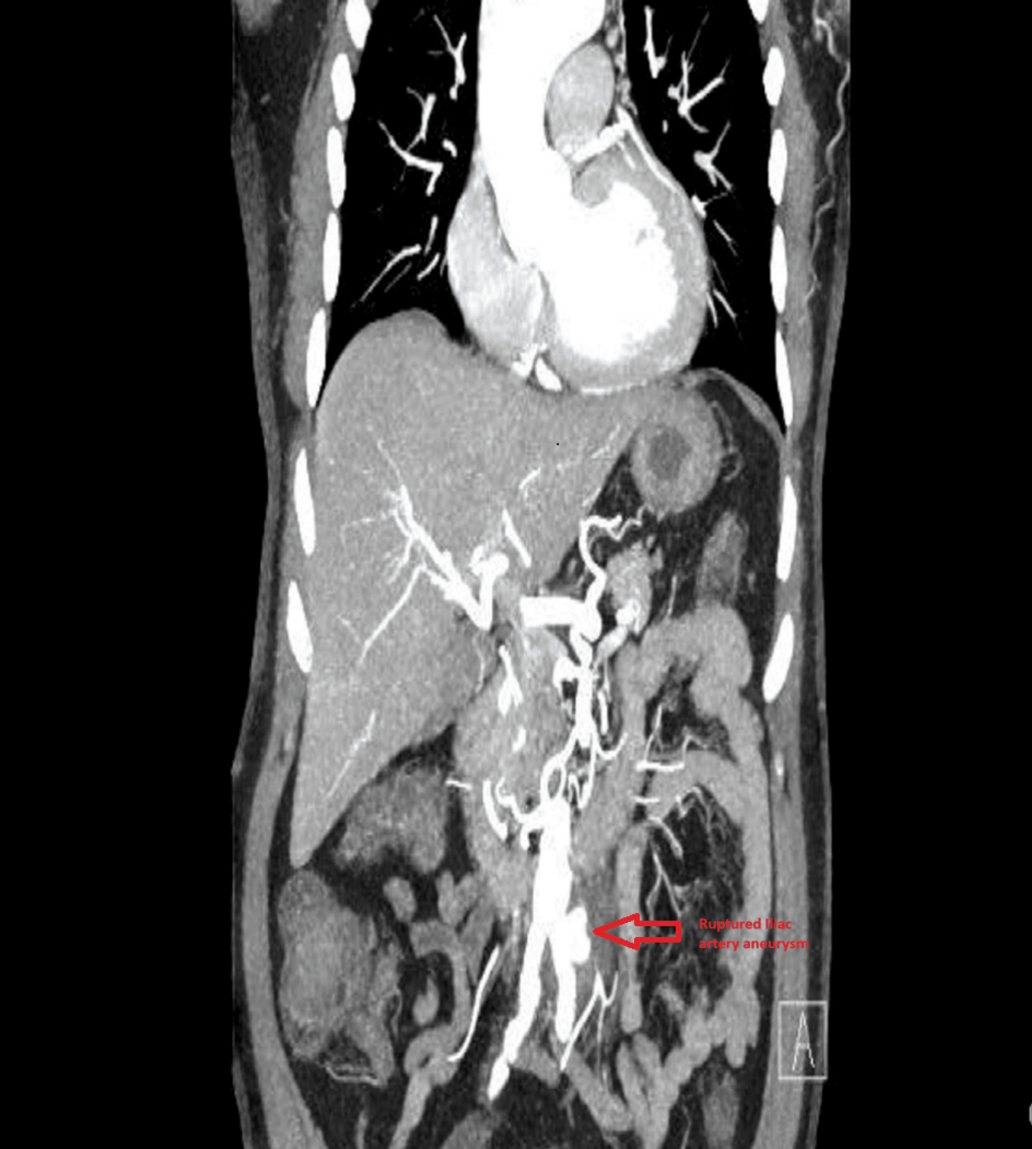
Ruptured iliac artery aneurysm

This patient was a 47-year-old man with a known medical history of IgG4-related disease and LVV. He presented to the emergency department in a state of confusion and hypotension, indicative of a critical hemodynamic compromise. On presentation, his blood pressure was 70/40 mmHg. He was transferred to the CT scan suite immediately for a CT angio. In addition to these symptoms, the patient also reported significant pain localized to the left renal region, raising concerns for a potential vascular or renal pathology. CT aortic angiogram was performed which showed a leaking aneurysm at the level of the left common iliac artery as shown in Figure 1.

A plan to perform damage control surgery was made to control bleeding but the patient did not pull through.

Case 2

**Figure 2:**
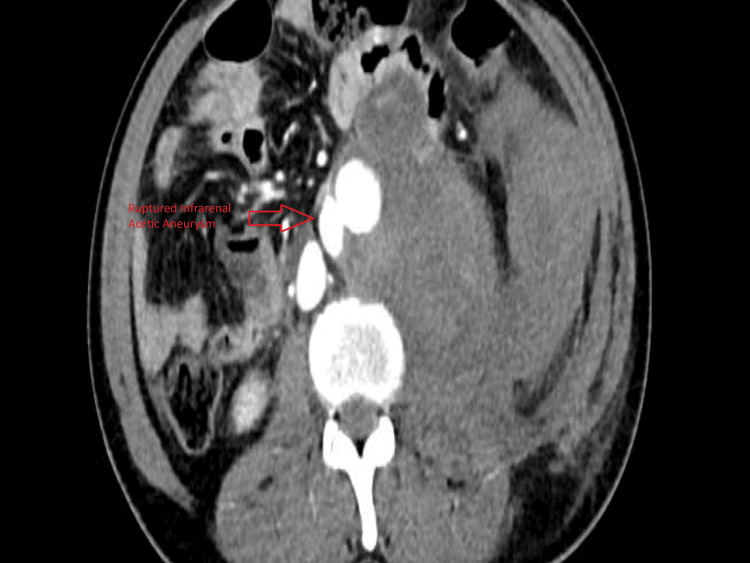
CT scan showing contrast extravasation in infrarenal abdominal aorta

The patient was a 42-year-old man who presented with a ruptured pseudoaneurysm. CT showed contrast leakage at the level of origin of the Inferior mesenteric artery and large retroperitoneal hematoma as shown in Figure 2.

The patient underwent surgical repair using a bi-iliac Dacron graft. After surgery, the patient was stable but had hypertension for which he was treated with medications: amlodipine, bisoprolol, and later on lisinopril. After the surgery, the patient still complained of left iliac region pain. CT contrast showed a large hematoma extending from the diaphragm to the groin region. The patient was treated conservatively with IV antibiotics. Vasculitis is suspected in this patient. A PET scan was performed but a final diagnosis could not be reached because changes in the scan could be attributed to postoperative changes also.

Case 3

**Figure 3:**
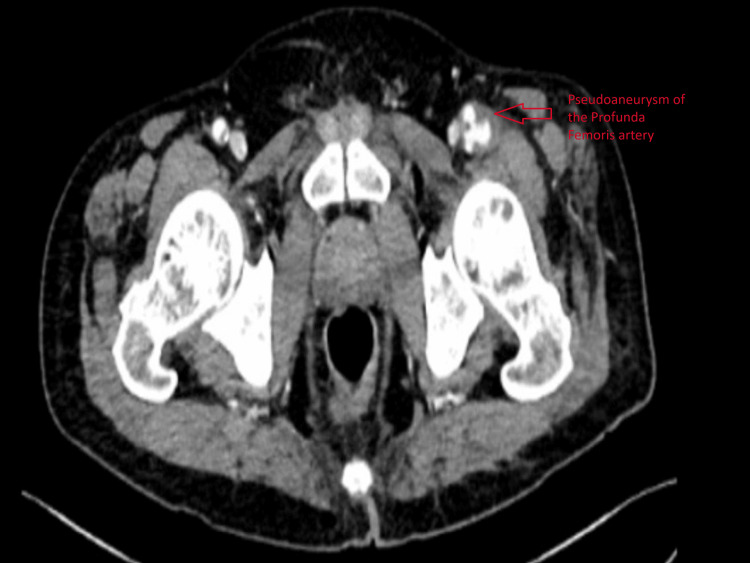
CT scan showing pseudoaneurysm of the profunda femoris artery

The patient was a 58-year-old man who initially presented in 2022 with left lower limb ischemia for which left femoral-popliteal bypass was done. He also had a history of melena, for which he was referred to gastroenterology. Colonoscopy showed a distorted caecum with deep ulcers. Biopsies were consistent with inflammation/ulceration. Scrotal ulcer biopsy showed dense acute inflammation with granulation tissue formation. Due to long-term history of oral ulcers and recent episodes of scrotal ulcers, rheumatology and dermatology were consulted. Syphilis was ruled out when the TPHA-Syphilis test was non-reactive. C-ANCA, P-ANCA, and ANA tests were negative. The patient was suspected to have Behçet’s disease and was started on colchicine, azathioprine, and steroids, to which he responded. He then started experiencing a sudden onset of pain in the left femoral region, along with swelling, which worsened on lying down. On examination, peripheral pulses were palpable, and noticeable swelling could be felt at the left femoral region, mildly tender, compressible, and pulsating. CT angiography was advised, which revealed a pseudo-aneurysm arising from the left profunda femoris artery just after its origin, along with a small outpouching arising from the left superficial femoral artery (Figure 3).

He was then planned for surgical intervention. He underwent ligation of the left profunda femoris artery at the origin of the feeding aneurysm, plus exploration of the aneurysm and closure of the remaining aneurysm.

Case 4

The patient, a 47-year-old man, had a medical history significant for hypertension, Behçet’s disease, and deep vein thrombosis (DVT). He was a chronic smoker and had been on anticoagulant therapy for the past four years.

The patient presented to the ED with complaints of lower abdominal discomfort, lower back pain, and constipation, persisting for two weeks. Although he reported a history of fever, it was undocumented. Upon admission, the patient’s vital signs were within normal limits: heart rate of 67 beats per minute, blood pressure of 128/87 mmHg, respiratory rate of 16 breaths per minute, and he was afebrile.

**Figure 4:**
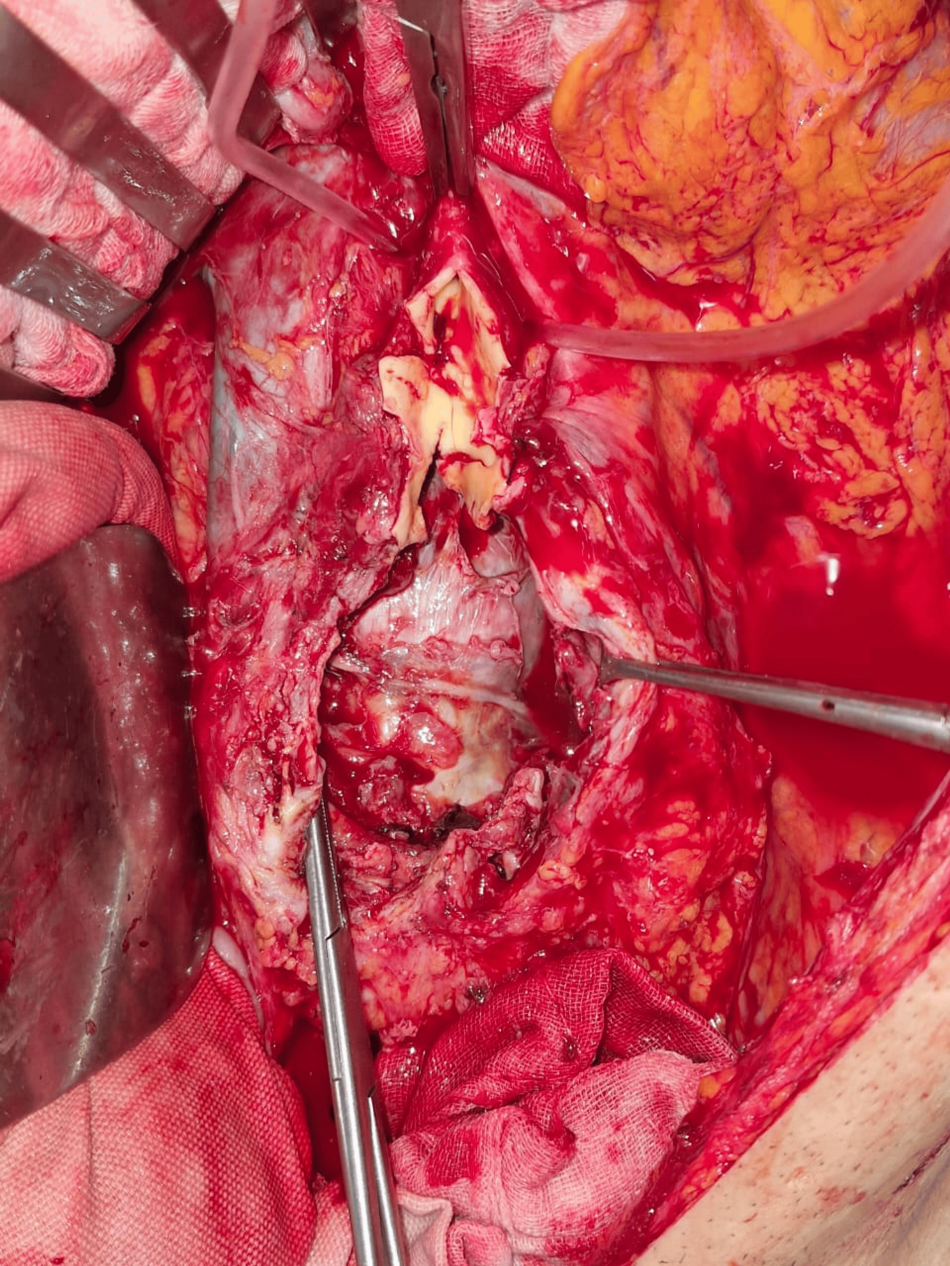
Surgery performed for the abdominal aortic aneurysm

In 2018, the patient had been diagnosed with an abdominal aortic aneurysm (AAA) through computed tomography (CT) angiography. A repeat CT angiography in the ED revealed a 5 cm infrarenal AAA, with symptoms suggestive of impending rupture. Given the risk, the patient was promptly taken to the operating room, where surgical repair was performed using a straight graft (Figure 4).

The postoperative course was uneventful, and the patient was discharged in stable condition on postoperative day nine.

The patient was diagnosed with Behçet’s disease at the age of 25 based on a history of oral and genital ulcers, for which he was treated with corticosteroids. In 2004, he developed uveitis, and his diagnosis was confirmed in 2008, leading to the initiation of azathioprine and corticosteroid therapy. More recently, the patient experienced recurrent episodes of DVT and was treated with rivaroxaban for one month.

## Discussion

Etiology of vascular aneurysms in Bechet’s disease is complex and many theories have been proposed. Literature suggests that immune reactions lead to the destruction of tunica media. An increase in the number of neutrophils and their adherence to endothelial cells has also been linked to vascular involvement in Bechet’s disease. Bechet’s disease is commonly characterized by recurrent oral and genital ulcers and relapsing ocular and skin lesions. LVV is not very common, but once the vasculature is involved, it contributes to the overall prognosis of the disease [10,11].

Management of aneurysms can be classified into either a conservative approach, open surgical, or endovascular repair. The conservative approach involves cholesterol lowering and anti-hypertensive drugs along with tobacco cessation. This is only adequate for patients who have an asymptomatic aneurysm [10,12].

Based on long-term follow-ups of eight patients, Okada et al. described the recurrence rate in the case of open surgical repair of the aneurysms [13]. Half of the patients needed another surgery, and two of them had a third one because of relapse. This has made the endovascular option a better choice of the two. Immune therapy in addition to endovascular treatment has less documented risk and better survival outcomes [14]. Despite hybrid procedures carrying less surgical trauma, some studies show that patients are equally likely to suffer a significant risk of anastomotic pseudoaneurysm to traditional open repair.

The endovascular method could lower the chances of aneurysm relapse and death after surgery, and surgery should not be done when Bechet’s disease is active. Controlling it with drugs in its active phase is essential for surgery. Endovascular operations are less invasive than open ones, but stents or stent grafts can cause inflammation, and mechanical irritation can lead to pseudoaneurysm after endovascular treatment. Using immunosuppressants before and after surgery for BD patients can help control inflammation and decrease the possibility of complications. The immunosuppressive treatment is based on the serial ESR measurement [15].

However, endovascular surgery is not always accessible or feasible, especially in case of complex or leaking aneurysms. In such situations, open surgery may be the only option to prevent life-threatening complications. Essential elements for early intervention include a detailed evaluation of the aneurysm's size, location, and associated vascular complications. Imaging studies such as CT angiography should be utilized for preoperative planning. An early diagnosis and assessment of vascular involvement are pivotal in determining the timing of intervention. Patients with rapidly enlarging, symptomatic, or ruptured aneurysms should be prioritized for early surgical intervention. Additionally, the decision to proceed with open surgery should be based on the patient’s overall condition, risk factors, and comorbidities. Patients should be stabilized with corticosteroids and other immunosuppressants to reduce the inflammatory burden and risk of complications during and after surgery. 

In our case series, open repair was done in all the cases. One patient did not survive the surgery, but the remaining patients are kept under close observation and on immunotherapy.

We recommend using immunosuppressants for a long duration. Except for urgent cases, the surgery should be delayed until the inflammation levels are reduced. Continuing corticosteroids and immunosuppressants for a minimum of two years after the surgery has been shown to have a beneficial effect [16]. Giving corticosteroids and immunosuppressants around the time of the surgery can also lower the risk of aneurysm recurrence [17].

## Conclusions

Vascular complications associated with Bechet’s disease can be life threatening. Therefore, we recommend involving vascular surgeons when rheumatologists encounter patients with Behcet’s disease. A regular follow-up with vascular surgeons can enable both the patient and the clinicians to identify any vascular complication developing and treat it before it can pose a threat to patient’s life. 
